# Compulsive sexual behavior, sexual functioning problems, and their linkages to substance use among German medical students: exploring the role of sex and trauma exposure

**DOI:** 10.3389/fpsyg.2024.1423690

**Published:** 2024-12-04

**Authors:** Dennis Jepsen, Tobias Luck, Christian Heckel, Jana Niemann, Kristina Winter, Stefan Watzke

**Affiliations:** ^1^Institute of Medical Sociology, Interdisciplinary Center of Health Sciences, Medical Faculty, Martin-Luther-University Halle-Wittenberg, Halle (Saale), Germany; ^2^Faculty of Applied Social Sciences, University of Applied Sciences Erfurt, Erfurt, Germany; ^3^Institute for Social Medicine, Rehabilitation Sciences and Health Services Research, Nordhausen University of Applied Science, Nordhausen, Germany; ^4^Department of Psychiatry, Psychotherapy, and Psychosomatics, University Hospital Halle, Halle (Saale), Germany

**Keywords:** adverse childhood experiences, addiction, childhood trauma, hypersexual behavior, hyposexual behavior, sexual dysfunction, post-traumatic stress

## Abstract

Sexual problems relevant to psychotherapy, such as compulsive sexual behavior (CSB) and sexual functioning problems (SFP), have been related to harmful substance use in several studies. Substance use is prevalent among medical students (MS) and is often considered a maladaptive coping strategy for stress, as well as a risk factor for mental health issues. Sexual problems and substance use share trauma exposure and post-traumatic symptoms as risk factors for their development. This study aimed to explore the interaction effects between problematic sexual behaviors, substance use, and trauma among German MS. A cross-sectional study (*n* = 359; 69% women, 29% men) was conducted using an online questionnaire. MS at a German university were recruited via email. CSB (CSBD-19), SFP (SBQ), harmful alcohol (AUDIT) and drug use (DAST), childhood trauma exposure (CTQ), and current post-traumatic symptoms (IES-R) were assessed. Multivariate linear and ordinal regressions, as well as path analyses, were conducted to investigate associations between the study variables. CSB was identified in 3% of all MS. The most commonly reported SFPs were decreased sexual desire and difficulties achieving orgasms among women and premature ejaculation among men. Higher CSBD scores were predicted by male sex, elevated AUDIT scores, and increased frequencies of hyperarousal (IES-R). Path analyses revealed associations between the severity of emotional/sexual abuse, the intensity of post-traumatic symptoms, and both CSBD and AUDIT scores. Among female MS, less severe emotional abuse and more severe physical abuse in childhood predicted higher frequencies of orgasmic difficulties. The frequency of SFPs was correlated with the use of benzodiazepines among female MS, with cannabis and MDMA/ecstasy among male MS, and with cocaine/crack, speed, and AUDIT among both sexes. No interaction effects were found between SFPs, substance use, or trauma-related factors in the path analyses. To some extent, there appears to be a relationship between substance use, childhood trauma exposure, and currently persisting post-traumatic symptoms with problematic sexual behaviors among MS. However, further research is required to explore these relationships in greater depth and to identify the underlying pathways. Mental health support measures should incorporate the factors of sexuality, substance use, and trauma while also exploring their relationships with workload, career-related anxieties, and other curriculum-related factors.

## 1 Introduction

### 1.1 Background

This study aimed to explore the trilateral relationships between problematic sexual behaviors, namely compulsive sexual behavior (CSB) and sexual functioning problems (SFP), substance use, and trauma-related factors among medical students (MS) in Germany. While regular substance use can lead to the development of sexual problems due to its effect on the body, depending on the type of substance consumed, substance use may also serve as a coping mechanism to deal with CSB and SFP and related consequences (Jepsen et al., 2023). Although all these factors have been individually investigated among German MS in previous studies, to the best of our knowledge, no study has examined them simultaneously.

Sexuality plays a significant role in many individuals’ lives, making it unsurprising that problematic sexual behaviors can contribute to persistent mental health challenges. “Emerging adulthood” defines the life stage between youth and adulthood (Arnett, 2014; Wood et al., 2018). In this phase of life, a variety of career opportunities and identities are explored while young adults gain more independence and experience less social control. Emerging adulthood is also a crucial life stage in terms of the exploration of individual sexuality (Vasilenko, 2021). Those who decide to go to university usually leave their parental home and start a campus life where they can explore the new environment on their own (Wood et al., 2018). During this transitional period, most emerging adults engage in sexual activities, typically in their late 30s or early 30s (Vasilenko, 2021). However, greater independence and less control have the potential to encourage hazardous health behaviors (Stone et al., 2012). This article focuses on two hazardous health behaviors among MS, who are a vulnerable group prone to persistent mental stress: problematic sexual behaviors and substance use. As we will learn later in this article, these two factors are strongly related to each other and can have a negative impact on wellbeing in general but also on MS.

#### 1.1.1 Problematic sexual behaviors

To further explore the link between problematic sexual behaviors and substance use, it is crucial to define when sexual behavior is considered problematic from a psychotherapeutic perspective. According to the current state of research, two dimensions of problematic sexual behaviors—compulsive sexual behavior (CSB) and sexual functioning problems (SFP)—seem to be relevant in research addressing harmful substance use behaviors.

CSB is defined by an extraordinarily high level of sexual activity and arousal, paired with the loss of control of sexual behaviors, resulting in psychological strain and functioning problems in important areas of life. It is characterized by behaviors such as excessive pornography consumption, masturbation, or sex dating (Grubbs et al., 2020). Conversely, SFP is defined as impaired or absent sexual desire, arousal, and activity, leading to clinically significant distress (World Health Organisation (WHO), 2024). There are various forms of SFP, including hypoactive sexual desire and impaired sexual arousal, solely to women’s problems with achieving orgasm and pain during sexual activity and solely to men’s erectile dysfunction and premature and delayed ejaculation (Briken et al., 2020; Jepsen et al., 2023). It is important to emphasize that fluctuations in the intensity of sexual arousal and frequency of sexual activity are normal. However, CSB and SFP are patterns that develop over time, with distress and loss of control being core characteristics that render them problematic from a psychotherapeutic standpoint.

Research on problematic sexual behaviors, such as CSB or SFP, among MS or physicians is scarce. Previous studies found SFP among 40–60% of female MS (Wallwiener et al., 2017; Shindel et al., 2008). In a study by Shindel et al. (2008), approximately 30% of male MS report erectile dysfunction and problems controlling ejaculation. No additional studies were found that focused on the SFP, and no study at all was identified that investigated CSB among MS.

#### 1.1.2 Substance use and sexual behavior

##### 1.1.2.1 General relationships between problematic sexual behaviors and substance use

Regular consumption of alcohol and illegal drugs is often associated with negative health outcomes, and sexual problems have received close attention in recent years (Jepsen et al., 2023). Substances such as alcohol, cannabis, and amphetamines can increase sexual arousal and activity in the short term, while more frequent or long-term use of psychotropic substances is associated with the development of SFPs (Jepsen et al., 2023; Krüger, 2020). In particular, the risky use of substances such as alcohol, cannabis, and cocaine has been associated with CSB (Ballester-Arnal et al., 2020; Jepsen et al., 2024). Harmful alcohol consumption is associated especially with vaginal pain during penetration and the absence of vaginal lubrication among women (Grover et al., 2014) and erectile dysfunction, premature ejaculation, and reduced sexual desire among men (Grover et al., 2014; Prabhakaran et al., 2018; Rohilla et al., 2020). Also, the regular use of other psychotropic substances, such as opioids, cocaine, cannabis, and methamphetamines, seems to be related to similar SFP (Grover et al., 2014; Diehl et al., 2016; Yee et al., 2016; Dolatshahi et al., 2016). In summary, risky or harmful substance use can have both excitatory effects, such as CSB, and inhibitory effects, such as SFP, on sexual behavior (Krüger, 2020). In this context, substances can be used to cope with problematic sexual behaviors that lead to impairments in important areas of life. Inversely, CSB can also arise as a coping mechanism for dealing with the consequences of problematic substance use in important areas of life. A third possibility is the development of SFP due to the physiological effects of regular or problematic substance use, which vary depending on the substance consumed (Hallinan, 2021; Jepsen et al., 2023; Palha and Esteves, 2008).

##### 1.1.2.2 Substance use among medical students and its relation to problematic sexual behaviors

Previous research has thoroughly examined the substance use behavior of MS and its associations with psychosocial risk factors. Most MS report alcohol consumption in the past year, while information about the prevalence of risky or harmful alcohol use varies between 9 and 34% (Bryl et al., 2020; Wang et al., 2023; Ayala et al., 2017; Voigt et al., 2009). Studies on licit and illicit substance use among German MS are rare and inconsistent in terms of investigated drugs and measurement approaches. Hoff et al. (2023) investigated *inter alia* the use of cannabis and alcohol among students in Germany across various fields of study. Approximately 40% of all students reported risky alcohol consumption, while MS showed a similar proportion compared to students in other fields. Cannabis was consumed by 11% of the total sample of students, whereas MS showed the lowest consumption proportion (8.3%). A sex effect was observed for cannabis consumption (and not for alcohol), with female students reporting lower consumption rates.

Various studies have revealed that alcohol, tobacco, and cannabis are the most commonly consumed substances among MS (Ayala et al., 2017; Roncero et al., 2015; Candido et al., 2018; Gupta et al., 2022). Substance use is more prevalent among male MS than among females, especially concerning alcohol and cannabis (Ayala et al., 2017; Voigt et al., 2009; Hoff et al., 2023; Roncero et al., 2015; Wilson et al., 2022). Further studies have identified a relationship between MS substance use and negative psychosocial and health-related outcomes. Hence, tranquilizer use appears to be linked to suicidal ideation (Keuch et al., 2023) and depressive symptoms (Pukas et al., 2022) among MS. Moreover, several studies suggest that physicians are a vulnerable group to developing substance use disorders (Beschoner et al., 2019), while it can be assumed that they use alcohol and drug abuse as maladaptive coping strategies to deal with occupational distress (Medisauskaite and Kamau, 2019; Vijendren et al., 2015). At the same time, the psychological distress level of MS is consistently higher than that of the general population (Dyrbye et al., 2006), which suggests a higher risk of implementing problematic substance use patterns as coping mechanisms.

In a study by Wallwiener et al. (2017), an association was found between alcohol consumption and the intensity of female sexual dysfunction in a sample of MS. No other study has examined the relationship between SFP and substance use among MS. To the best of our knowledge, no research investigated the relationship between CSB and substance use among MS. However, MS and physicians comprise vulnerable groups for developing harmful substance use patterns and their resulting psychosocial consequences (Dyrbye et al., 2006). Thus, it seems beneficial to explore associations with problematic sexual behavior as potential risk factors for developing and maintaining harmful substance use patterns.

#### 1.1.3 Trauma exposure and post-traumatic stress are common risk factors

Traumatic experiences comprise an often-identified risk factor for CSB (von Franqué and Briken, 2018), SFP (Lalchandani et al., 2020; Sack and Büttner, 2014), and substance abuse and disorders (Moustafa et al., 2021; Pace et al., 2022). It is essential to consider these factors while exploring the relationship between problematic sexual behaviors and substance use. Childhood maltreatment by primary attachment figures, including emotional, physical, and sexual abuse, as well as emotional and physical neglect, can lead to various psychosocial and health-related consequences (Gilbert et al., 2009), including post-traumatic symptoms. According to ICD-11 PTSD is characterized by three primary symptoms: intrusions (e.g., flashbacks or nightmares), avoidance (e.g., steering clear of activities or situations that trigger traumatic memories), and hyperarousal (a persistent sense of threat, leading to hypervigilance and related symptoms) (World Health Organisation (WHO), 2024).

The few studies that have examined childhood trauma exposure (CTE) among MS vary regarding identified prevalence and measures used. According to a systematic review by King et al. (2017), the prevalence of physical abuse is 5 to 65%. Emotional trauma is approximately 4%, sexual abuse is 3 to 13%, and general trauma is 13% among MS. In a study by Ignatova et al. (2022), most reported general trauma exposures of MS were witnessing violence, family mental illness, serious personal injuries, or illness of a parent or friend, while at least one type of general trauma was reported by around 80%. In the same study, correlations between CTE among MS and depression, anxiety, and compulsive symptoms were found, as well as with alexithymia and subjective lower quality of life (Ignatova et al., 2022). Furthermore, a higher risk of burnout is considered a suspected negative health outcome related to trauma exposure among MS (Sciolla et al., 2019). Because of the psychosocial consequences that CTE can have on individuals in general, as well as MS specifically, it is important to consider CTE in investigating the relationships between problematic sexual behaviors and substance use.

### 1.2 Objectives

Considering the current state of research, data on problematic sexual behaviors such as CSB and SFP, as well as related factors concerning mental health among MS, are scarce. Notably, several studies investigated substance use among MS, few explored SFP and CTE, and none focused on CSB among MS in Germany or in general. Further, to the best of our knowledge, no study has addressed trilateral associations between problematic sexual behavior, substance use, and CTE. This study aims to fill these research gaps by focusing on the following objectives:

1. Providing a first overview of frequencies of CSB and SFP among MS.
2. Identifying predictors of CSB and SFP among MS related to substance use and trauma.
3. Investigating the interaction effects between CSB, SFP, substance use, and trauma-related factors.

## 2 Materials and methods

### 2.1 Study design and procedure

Data were collected online through a cross-sectional explorative study between May and December 2023. The questionnaire was developed using the software *RedCap (Research Electronic Data Caption)*. The survey was conducted among MS at a mid-sized university in Germany. Students were invited to participate voluntarily by their lecturers during courses and through email, with all data collected anonymously. The study was conducted in accordance with the Declaration of Helsinki (World Medical Association (WMA), 2022).

### 2.2 Measures

#### 2.2.1 Compulsive sexual behavior and sexual functioning problems

CSB was measured via the German version of the Compulsive Sexual Behavior Scale (CSBD-19) (Bőthe et al., 2020), which includes 19 five-pointed ordinal-scaled items (answer options between *1 = not agree at all* and *4 = totally agree*) based on the ICD-11 diagnosis of compulsive sexual behavior disorder (CSBD). The scale consists of five subscales (control, salience, relapse, dissatisfaction, and negative consequences). The cut-off value of the sum score is ≥50, indicating a prevalent CSBD (Bőthe et al., 2020). The internal consistency was excellent (Cronbach’s *α* = 0.926).

Indications for SFP were measured using the German version of the Sexual Behavior Questionnaire (SBQ-G) (Müller, 2016), originally invented by Macdonald et al. (2003). The scale includes four-pointed ordinal-scaled items addressing the frequency of occurring problems concerning desire for sex, capability for sexual arousal, capability for sexual enjoyment, satisfaction with sexual life, and orgasm within the last 3 months. It measures several facets of SFP via six items concerning sexual behavior applied to all sexes and five or six additional items regarding sex-specific behaviors. For women, items addressed orgasmic dysfunction and pain during penetration, and for men, erectile dysfunction, premature, and delayed ejaculation. Values of 0 and 1 on the respective ordinal-scaled items indicate SFP (Müller, 2016).

#### 2.2.2 Substance use

The German version of the Alcohol Use Disorder Identification Test (AUDIT) was used to measure alcohol consumption habits via three subscales (alcohol consumption, dependence, and alcohol-related consequences), consisting of 3- to 5-pointed Likert-scaled items. Depending on the calculated sum score, alcohol use habits can be classified as abstinence/low-risk use (values from 0 to 7), risky use (values from 8 to 15), harmful use (values from 16 to 19), and chronic alcoholism (values from 20 to 40) (Babor and Robaina, 2016). The internal consistency was good (Cronbach’s *α* = 0.803).

Moreover, the participants were asked which illegal drugs they use/have used and how regularly. The Drug Abuse Screening Test (DAST-10), consisting of ten dichotomous items (answer options *0 = no* and *1 = yes*), was used to identify problematic consumption of illegal drugs within the past 12 months. Based on the determined sum scores, drug use can be classified as low/moderate (values from 1 to 5), substantial (values from 6 to 8), and severe level (values from 9 to 10) (National Institute on Drug Abuse, 2014; Yudko et al., 2007). The scale was translated into German via forward-backward translation (Acquadro et al., 2008) since no German version of the DAST currently exists. The internal consistency of the DAST was good (Cronbach’s *α* = 0.883).

The consumption frequencies of illicit drugs—including cannabis; amphetamines (MDMA/ecstasy, mephedrone, crystal meth, and Ritalin); hallucinogens (LSD, ketamine, other hallucinogens such as mushrooms/mescaline); cocaine/crack; opiates/opioids (heroin, fentanyl, and methadone), tranquilizers (barbiturates, benzodiazepines, and z-substances); and other illicit substances—were measured using eight-point ordinal scale items, with response options ranging from 1 (never) to 8 (every day).

#### 2.2.3 Trauma exposure and post-traumatic stress

The German version (Klinitzke et al., 2012) of the Childhood Trauma Questionnaire (CTQ) by Bernstein et al. (1994) was utilized to measure trauma exposure during childhood and adolescence. The inventory consists of 28 five-pointed ordinal-scaled items, divided into five subscales, measuring the severity of emotional abuse (Cronbach’s α = 0.846), physical abuse (Cronbach’s α = 0.774), sexual abuse (Cronbach’s α = 0.911), emotional neglect (Cronbach’s α = 0.893), and physical neglect (Cronbach’s α = 0.465) using sum scores, respectively.

To assess currently occurring symptoms related to post-traumatic stress, the Impact of Event Scale (revised; IES-R) by Weiss and Marmar (1996) was used. It consists of 22 four-pointed ordinal items and three subscales, measuring the frequencies of currently occurring post-traumatic symptoms: intrusions (Cronbach’s α = 0.930), avoidance (Cronbach’s α = 0.923), and hyperarousal (Cronbach’s α = 0.892) with sum scores, respectively. In addition, the total IES-R score (Cronbach’s α = 0.962) was used for the data analyses.

#### 2.2.4 Control variables

Participants were asked which semester they were currently studying. Sociodemographic information was collected, including age, sex, gender identity, size of current place of residence, marital status, religious affiliation, highest educational degree, and occupational status. Data on somatic and psychiatric conditions, medications, and relevant medical histories of family members and other important persons of participants were collected, as compulsive sexual behavior or sexual functioning problems may be related to other health-related factors beyond substance use.

#### 2.2.5 Data analyses

Data analysis was conducted using SPSS version 28 and R. Mean value comparisons by sex were calculated using *t*-tests for independent samples (for the metric scales: AUDIT, DAST, CSBD, CTQ subscales, and IES-R subscales) and U-tests (for all SBQ items), which were ordinal-scaled.

*T*-tests were conducted if the variables’ kurtosis and skewness levels fell within the acceptable ranges of −/+ 7 for kurtosis and −/+2 for skewness, indicating normal distribution (Hair et al., 2010). Kurtosis and skewness for all subscales can be found in Supplementary Table S1. Levene’s test was used to prove the homogeneity of variances.

In the first step, correlations and Cramer’s *V* (*CV*) were calculated for all relevant variables. Stepwise multiple regression was conducted, with the CSBD score as a dependent variable and all other (control) variables that showed significant correlations with the CSBD score. AUDIT, DAST, substance consumption frequency, CTQ-, and IES-R subscales were included in the first block of the stepwise regression, and control variables were added in the second block. Normal distribution of the residuals was tested using the Breusch-Pagan test to see if the residuals were normally distributed. If this was not the case, the White test was applied accordingly. Variance inflation factors (VIF) were used to check for multicollinearity, which was assumed for values of >10 (Marcoulides and Raykov, 2019). Ordinal regression models were calculated with every investigated occurrence frequency of SFP (SBQ) as the respective dependent variable and the same potential predictors.

To better interpret the results of correlation and regression analyses, a path analysis with maximum-likelihood estimation was conducted using *R Studio*, incorporating CTQ subscales (*Z_1_*), IES-R subscales (*Z_2_*), substance use (Z*_3_*), and CSBD scores/frequencies of SFPs (*Z_4_* Model fit was evaluated using chi-square), root mean square error of approximation (RMSEA), and comparative fit index (Backhaus et al., 2016; Kenny et al., 2015). Given that sample size and degrees of freedom can influence the performance of RMSEA, we examined its 90% confidence interval according to the recommendations of Kenny et al. (2015). For RMSEA, values below 0.08 are deemed acceptable (Browne and Cudeck, 1993; Kenny et al., 2015; MacCallum et al., 1996), while CFI values above 0.90 indicate a good fit (Backhaus et al., 2016).

## 3 Results

### 3.1 Descriptive results

#### 3.1.1 Sociodemographic information and health status

The survey was started by *n* = 445 MS, but after data cleaning and removing all cases of people who did not answer the items about problematic sexual behaviors, the final sample size was reduced to *n* = 359. Most participants were in their third semester (32.0%, *n* = 115), followed by the ninth (23.1%, *n* = 83) and fifth semester (21.2%, *n* = 76). The average age was *M* = 23.6 (*SD* = 3.4). Female gender identity was reported by 69.4% (*n* = 249), male gender identity by 29.0% (*n* = 104), non-binary by 0.8% (*n* = 3), and another not specified gender identity by 0.3% (*n* = 1). However, 64.3% (*n* = 231) stated female sex and 26.5% (*n* = 95) male sex. The gender distribution of the sample is roughly consistent with the current national comparison in Germany (64.4% female and 35.6% male) (Statistisches Bundesamt, 2024). Approximately one-fifth of the sample (21.7%, *n* = 78) reported somatic diseases (especially respiratory and dermatological diseases), and 18.4% (*n* = 66) stated a psychiatric disorder, with depression being the most frequently mentioned (*n* = 41). See Table 1 for the frequencies of all the measured socio-demographic information.

**Table 1:** Sociodemographic information.

	Answer options	*n* (%)
Legal gender	Female	251 (69.9)
	Male	107 (29.8)
	*Missing*	*1 (0.3)*
Gender identity	Female	249 (69.4)
	Male	104 (29.0)
	Non-binary	3 (0.8)
	Other	1 (0.3)
	*Missing*	*2 (0.6)*
Sex	Female	231 (64.3)
	Male	95 (26.3)
	Intersex	0 (0.0)
	*Missing*	*33 (9.2)*
Size of current place of residence	Under 5,000 inhabitants	8 (2.2)
	Under 20,000 inhabitants	4 (1.1)
	Under 100,000 inhabitants	14 (3.9)
	As of 100,000 inhabitants	327 (91.1)
	1,000,000 and more inhabitants	5 (1.4)
	*Missing*	*1 (0.3)*
Relationship status	Single	127 (35.4)
	In a relationship (not married)	210 (58.5)
	Married	20 (5.6)
	Divorced	1 (0.3)
	*Missing*	*1 (0.3)*
Religious affiliation	None	195 (54.3)
	Evangelistic	104 (29.0)
	Catholic	43 (12.0)
	Muslim	7 (1.9)
	Jewish	1 (0.3)
	Other	8 (2.2)
	*Missing*	*1 (0.3)*
Employment	None	185 (51.5)
	Part-time	145 (40.4)
	Full-time	27 (7.5)
	*Missing*	*2 (0.6)*
Receipt of educational support	No	263 (73.3)
	Yes	94 (26.2)
	*Missing*	*2 (0.6)*

#### 3.1.2 (Problematic) sexual behavior

**Figure 1:**
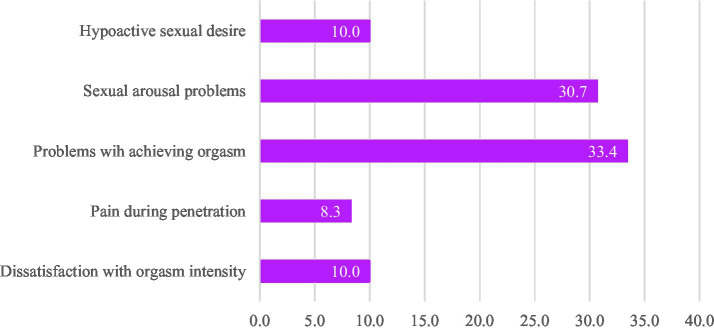
Frequency of sexual functioning problems among female medical students (%). Hypoactive desire was indicated if the answer option “I have no desire for sex” was chosen. Sexual arousal problems were indicated if the answer options “I never get aroused” or “I laboriously get aroused” were chosen. Problems with achieving orgasm were indicated if the answer options “I never have an orgasm” or “I occasionally have an orgasm” were chosen. Pain during penetration was indicated if the answer options “I always have pain” and “I often have pain” were chosen. Dissatisfaction with orgasm intensity was indicated if the answer options “I am not satisfied at all” or “I am somewhat satisfied” were chosen.

**Figure 2:**
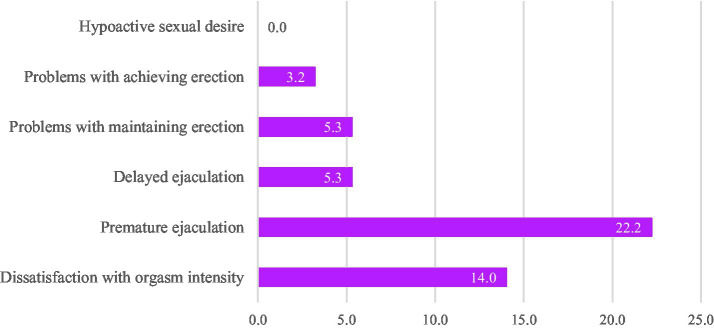
Frequency of sexual functioning problems among male medical students. Hypoactive desire was indicated if the answer option “I have no desire for sex” was chosen. Sexual arousal problems were indicated if the answer options “I never get aroused” or “I laboriously get aroused” were chosen. Problems with achieving erection were indicated if the answer options “I never get an erection” or “I occasionally get an erection” were chosen. Problems with maintaining erection were indicated if the answer options “I am never able to maintain an erection” I am occasionally able to maintain an erection” were chosen. Delayed ejaculation was indicated if the answer options “My ejaculation is always delayed” and “My ejaculation is often delayed” were chosen. Premature ejaculation was indicated if the answer options I always *cum* too early” and “I often *cum* too early” were chosen. Dissatisfaction with orgasm was indicated if the answer options “I am not satisfied at all” or “I am somewhat satisfied” were chosen.

The CSBD cut-off value of ≥50 was reached by 3.1% (*n* = 11, *n_female_* = 6, *n_male_* = 5). The mean CSBD score was 25.6 (*SD* = 9.1). No significant mean value difference among CSBD scores by sex was found. As presented in Table 2, according to the U-test results, female participants reported slightly, yet significantly, lower frequencies of sexual desire, masturbation, and enjoyment of sex/masturbation than male participants. However, they also reported to be slightly more satisfied with their overall sex life. The most common SFP reported by men was premature ejaculation, affecting 22.2%. Conversely, the most frequent SFPs among female MS were sexual arousal and orgasmic problems, reported by 30.7 and 33.4%, respectively. The frequencies of all the investigated sexual functioning problems are shown in Figures 1, 2.

**Table 2:** Sexual behavior and satisfaction of medical students stratified by sex.

	*n* (%)			Mean value comparison (U-Test)
	Total (*n* = 359)	Female (*n* = 251)	Male (*n* = 95)	Female vs. Male
How often would you like to have sex				
Never	23 (6.4)	23 (10.0)	–	M_f_ = 2.8 (SD = 0.81)
Less than once a week	43 (12.0)	38 (16.5)	4 (3.7)	M_m_ = 3.5 (SD = 0.58)
1–3 times a week	180 (50.1)	136 (58.9)	44 (46.3)	**U = 5971.50**
More than 3 times a week	80 (22.3)	33 (14.3)	47 (49.5)	**Z = −7.13**
*Missing/ not specified*	*33 (9.2)*	*1 (0.4)*	–	**p < 0.001**
How often do you have sex?				
Never	65 (18.1)	50 (21.6)	15 (15.8)	M_f_ = 2.3 (SD = 0.90)
Less than once a week	109 (30.4)	71 (30.7)	38 (40.0)	M_m_ = 2.4 (SD = 0.89)
1–3 times a week	123 (34.3)	91 (39.4)	31 (32.6)	U = 10544.00
More than 3 times a week	28 (7.8)	17 (7.4)	11 (11.6)	Z = −0.46
*Missing/ not specified*	*34 (9.5)*	*2 (0.9)*	–	*p* = 0.65
How often do you masturbate?				
Never	41 (11.4)	38 (16.5)	3 (3.2)	M_f_ = 2.4 (SD = 0.87)
Less than once a week	98 (27.3)	88 (38.1)	10 (10.5)	M_m_ = 3.3 (SD = 0.78)
1–3 times a week	122 (34.0)	82 (35.5)	40 (42.1)	**U = 5021.00**
More than 3 times a week	63 (17.5)	21 (9.1)	42 (44.2)	**Z = −8.00**
*Missing/ not specified*	*35 (9.7)*	*2 (0.9)*	–	**p < 0.001**
How often do you enjoy sex/ masturbation?				
Never	1 (0.3)	1 (0.4)	–	M_f_ = 3.1 (SD = 0.65)
Occasionally	47 (13.1)	38 (16.5)	9 (9.5)	M_m_ = 3.4 (SD = 0.65)
Often	173 (48.2)	131 (56.7)	42 (44.2)	**U = 7798.50**
Always	94 (26.2)	51 (22.1)	43 (45.3)	**Z = −3.90**
*Missing/not specified*	*44 (12.3)*	*10 (4.3)*	*1 (1.1)*	**p < 0.001**
How satisfied are you with your sexual life (including sex and masturbation)				
Never satisfied	10 (2.8)	3 (1.3)	7 (7.4)	M_f_ = 2.9 (SD = 0.68)
Occasionally satisfied	86 (24.0)	58 (25.1)	28 (29.5)	M_m_ = 2.6 (SD = 0.74)
Often satisfied	176 (49.0)	124 (53.7)	52 (54.7)	**U = 8918.50**
Always satisfied	42 (11.7)	34 (14.7)	8 (8.4)	**Z = −2.24**
*Missing/not specified*	*45 (12.5)*	*12 (5.2)*	*–*	**p = 0.03**

M, mean value; f, female; m, male; SD, standard deviation. Regarding the items measuring sexual dysfunction via SBQ, sex was measured via the item “Which sex characteristics do you have?” Answer options: Sex characteristics of a biological woman (vulva, vagina)/ of a biological man (penis, testicles)/ I am intersex (which was reported by none of the participants). Significant results are formatted in bold.

#### 3.1.3 Substance use

According to the AUDIT score, alcohol consumption was classified as unproblematic by most of the participants (66.0%, *n* = 237, *n_female_* = 185, *n_male_* = 52). An indication of a harmful consumption pattern was identified in 30.4% (*n* = 109, *n_female_* = 55, *n_male_* = 54), while 6.4% (*n* = 23, *n_female_* = 12, *n_male_* = 11) reached a critical value, indicating a requirement for professional consultation with great probability. A significantly higher AUDIT Score was identified among male MS (*M* = 7.9, *SD* = 4.8) compared to female MS (*M* = 5.5, *SD* = 4.3), *t*(344) = −4.72, *p* < 0.001, *d* = 0.53 (equal variances assumed, Levene’s test *p* = 0.06).

Regarding DAST score, an indication for harmful drug consumption was identified in 4.2% (*n* = 15, *n_female_* = 10, *n_male_* = 5) of the sample, whereas among 53.8% (*n* = 193, *n_female_* = 119, *n_male_* = 74), drug use could be determined as unproblematic (included were all participants who stated they used drugs at least once in their lifetime). The mean value comparison of the DAST score by gender identity showed insignificant results, *t*(206) = −0.55, *p* = 0.58. The consumption frequencies of all the investigated substances are presented in Table 3. Cannabis was the most consumed illicit drug within the sample, followed by ecstasy/MDMA, speed, and cocaine/crack. Male MS used cannabis (*CV* = 0.31, *p* = 0.01) more often than female MS, and no other sex differences were found concerning the frequency of illegal drug consumption. A significant relationship was found between indications of harmful alcohol consumption and drug use (*CV* = 0.25, *p* < 0.001).

**Table 3:** Consumption frequency of illegal drugs, *n* (%).

	Cannabis	Ecstasy/MDMA	Speed	Cocaine/ crack	LSD	Other hallucinogens	Benzodiazepine	Ketamine	Ritalin
Already consumed	218 (60.7)	51 (14.2)	39 (10.9)	42 (11.7)	25 (7.0)	26 (7.2)	20 (5.6)	17 (4.7)	16 (4.5)
Already consumed it, but not anymore	103 (28.7)	24 (6.7)	18 (5.0)	20 (5.6)	13 (3.6)	14 (3.9)	11 (3.1)	10 (2.8)	11 (3.1)
Once a year	27 (7.5)	9 (2.5)	10 (2.8)	6 (1.7)	6 (1.7)	10 (2.8)	–	1 (0.3)	1 (0.3)
2–4 times a year	49 (13.6)	13 (3.6)	7 (1.9)	13 (3.6)	1 (0.3)	1 (0.3)	5 (1.4)	3 (0.8)	3 (0.8)
Once a month	18 (5.0)	4 (1.1)	2 (0.6)	1 (0.3)	3 (0.8)	1 (0.3)	1 (0.3)	2 (0.6)	1 (0.3)
Once in 2 weeks	5 (1.4)	1 (0.3)	1 (0.3)	1 (0.3)	2 (0.6)	–	1 (0.3)	1 (0.3)	–
Once a week	6 (1.7)	–	1 (0.3)	–	–	–	2 (0.6)	–	–
2–4 times a week	8 (2.2)	–	–	1 (0.3)	–	–	–	–	–
Everyday	2 (0.6)	–	–	–	–	–	–	–	–
Never consumed	119 (33.1)	266 (74.1)	275 (76.6)	277 (77.2)	289 (80.5)	283 (78.8)	289 (80.5)	298 (83.0)	298 (83.0)
*Missing*	*22 (6.1)*	*42 (11.7)*	*45 (12.5)*	*40 (11.1)*	*45 (12.5)*	*50 (13.9)*	*50 (13.9)*	*44 (12.3)*	*45 (12.5)*

MDMA, 3,4-Methylenedioxymethamphetamine; LSD, lysergic acid diethylamide. Following drugs were part of the survey but are not included in the table because only a small number of participants reported that they had consumed them already: Z-substances *n* = 11, GHB/ liquid ecstasy *n* = 7, mephedrone *n* = 7, fentanyl *n* = 4, barbiturates *n* = 3, heroin *n* = 3, methadone *n* = 3, methamphetamine/crystal meth *n* = 5.

#### 3.1.4 Childhood trauma exposure and post-traumatic symptoms

**Figure 3:**
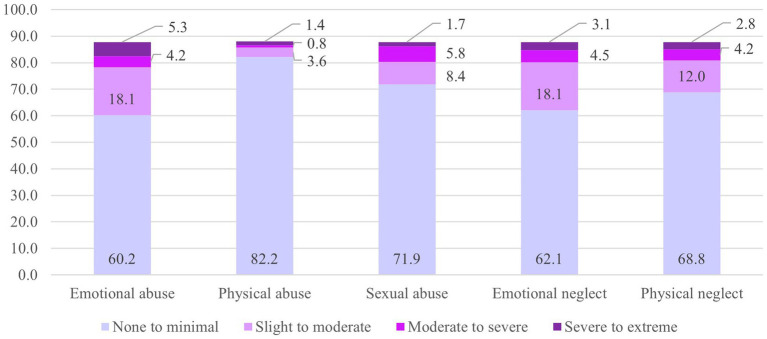
Severity of childhood trauma exposure (%). CTQ, Childhood trauma questionnaire; f, female; m, male. SD, standard deviation. Regarding the items measuring sexual dysfunction via SBQ, sex was measured via the item “Which sex characteristics do you have?” Answer options: Sex characteristics of a biological woman (vulva, vagina)/ of a biological man (penis, testicles)/ I am intersex (which was reported by none of the participants).

The frequencies of traumatic exposure in the sample stratified by sex are presented in Figure 3. The severity of emotional abuse was significantly higher for female MS (*t*(316) = 2.10, *p* = 0.04, *d* = 0.26) than for male MS, with a small effect size (equal variances assumed, Levene’s test *p* = 0.13). No other significant mean value comparisons were observed. No sex differences were found in the severity of currently occurring post-traumatic symptoms.

### 3.2 Correlative results with problematic sexual behaviors as dependent variables

Tables 4, 5 show the correlations of CSBD score/SBQ subscale scores and variables measuring facets of substance use, trauma exposition, and current post-traumatic stress symptoms.

**Table 4:** Correlationships between compulsive sexual behavior score and independent variables (Pearson’s product–moment coefficient, *r*).

	CSBD score	AUDIT score	DAST score	Severity of emotional abuse^a^	The severity of physical abuse^a^	The severity of sexual abuse^a^	Severity of emotional neglect^a^	Severity of physical neglect^a^	IES-R score	Intrusions^b^	Avoidance^b^	Hyperarousal^b^
CSBD score	1.00	0.28***	0.05	0.24***	0.25***	0.09	0.23***	0.21***	0.27***	0.19*	0.25**	0.32***
AUDIT score		1.00	0.40***	0.07	0.07	0.04	0.04	0.07	0.06	−0.03	0.10	0.05
DAST score			1.00	0.17*	0.10	−0.03	0.15*	0.01	0.13	0.12	0.10	0.11
Severity of emotional abuse^a^				1.00	0.60***	0.34***	0.76***	0.42***	0.53***	0.48***	0.45***	0.52***
The severity of physical abuse^a^					1.00	0.30***	0.55***	0.36***	0.35***	0.37***	0.20*	0.41***
The severity of sexual abuse^a^						1.00	0.32***	0.23***	0.52***	0.44***	0.53***	0.42***
Severity of emotional neglect^a^							1.00	0.56***	0.48***	0.42***	0.37***	0.54***
Severity of physical neglect^a^								1.00	0.28***	0.22**	0.20**	0.33***
IES-R score									1.00	0.93***	0.90***	0.89***
Intrusions^b^										1.00	0.74***	0.81***
Avoidance^b^											1.00	0.66***
Hyperarousal^b^												1.00

CSBD, Compulsive Sexual Behavior Scale (CSBD-19); AUDIT, Alcohol Use Disorder Identification Test; DAST, Drug Abuse Screening Test. *** = *p* < 0.001. ** = *p* < 0.01. * = *p* < 0.05.

a Subscales of Childhood Trauma Questionnaire (CTQ).

b Subscales of Impact of Event Scale (revised; IES-R).

**Table 5:** Correlationships between sexual behavior/dysfunction and independent variables (Spearman’s rank correlation coefficient, *r*).

	CSBD score	AUDIT score	DAST score	Severity of emotional abuse^a^	The severity of physical abuse^a^	The severity of sexual abuse^a^	Severity of emotional neglect^a^	Severity of physical neglect^a^	IES-R score	Intrusions^b^	Avoidance^b^	Hyperarousal^b^
General sexual behavior^c^												
Sexual desire	0.43***	0.30***	0.09	0.00	0.13*	−0.02	−0.07	−0.01	−0.03	−0.05	−0.01	−0.03
Intercourse frequency	0.15**	0.17**	0.03	−0.06	0.07	0.02	−0.09	−0.02	0.01	0.06	0.05	−0.01
Masturbation frequency	0.29***	0.18**	0.20**	0.03	0.10	−0.04	0.04	0.06	0.09	0.06	0.06	0.07
The ability of sexual arousal	0.32***	0.19***	0.00	0.00	0.01	−0.12*	−0.13*	−0.10	−0.04	−0.07	−0.01	−0.04
Ability to achieve sexual enjoyment	0.01	−0.01	0.05	−0.13*	−0.08	−0.17**	−0.21***	−0.18**	−0.21**	−0.15	−0.22**	−0.20*
Satisfaction with sexual life	−0.22***	−0.10	−0.15*	−0.10	−0.12*	−0.07	−0.24***	−0.13*	−0.18*	−0.15	−0.12	−0.23**
Female sexual function												
Ease of reaching orgasm	0.00	−0.06	0.06	0.07	0.00	0.00	−0.02	−0.06	0.12	0.13	0.15	0.05
Satisfaction with orgasm intensity	0.05	−0.10	0.07	0.11	0.01	0.01	−0.06	−0.06	0.05	0.09	0.10	−0.03
Pain during penetration^d^	−0.15*	−0.04	−0.37***	−0.08	−0.14*	−0.13	−0.08	−0.04	−0.18*	−0.18	−0.19*	−0.10
Male sexual function												
Ability to get an erection	−0.11	−0.08	−0.15	−0.10	−0.31**	−0.15	0.02	−0.03	−0.09	−0.01	−0.10	−0.12
Ability to maintain erection	−0.12	0.04	−0.02	−0.13	−0.21*	−0.04	−0.12	−0.15	−0.10	−0.01	−0.18	−0.04
Frequency of delayed ejaculation	−0.28**	−0.17	0.09	−0.14	−0.06	−0.07	−0.04	−0.11	−0.19	−0.19	−0.11	−0.21
Frequency of premature ejaculation	−0.09	−0.13	−0.15	−0.05	−0.03	0.11	−0.13	−0.14	−0.12	−0.24	−0.19	−0.18
Satisfaction with orgasm intensity	−0.16	−0.21*	−0.19	−0.26*	−0.07	0.00	−0.35***	−0.23*	−0.22	−0.27	−0.22	−0.26

CSBD, Compulsive Sexual Behavior Scale (CSBD-19); AUDIT, Alcohol Use Disorder Identification Test; DAST, Drug Abuse Screening Test. *** = *p* < 0.001. ** = *p* < 0.01. * = *p* < 0.05.

a Subscales of Childhood Trauma Questionnaire (CTQ).

b Subscales of Impact of Event Scale (revised; IES-R).

c Correlationships were calculated within the total sample.

d Higher values indicate less frequent pain during penetration.

The other correlation analyses were calculated as sex-stratified.

#### 3.2.1 Correlations between problematic sexual behaviors and substance use frequency

No significant correlations were found between the CSBD score and the consumption frequencies of alcohol and illicit drugs.

Among female MS, the frequency of sexual desire correlated with alcohol consumption frequency (*r* = 0.22, *p* < 0.001). The frequency of sexual activity was related to the frequency of consumption of hallucinogenic substances (except LSD; *r* = 0.72, *p* = 0.01). Masturbation frequency was negatively correlated with ketamine (*r* = −0.76, *p* = 0.05), and speed (*r* = −0.50, *p* = 0.04) consumption frequency. The frequencies of benzodiazepine use (*r* = −0.88, *p* < 0.001) and cocaine/crack use (*r* = −0.50, *p* = 0.03) were associated with a decreased ability to enjoy orgasm. Additionally, a higher frequency of speed consumption was associated with decreased ability to become sexually aroused (*r* = −0.60, *p* = 0.01) and reach orgasm (*r* = −0.49, *p* = 0.05), as well as decreased satisfaction with the intensity of orgasm (*r* = −0.58, *p* = 0.02). The frequency of pain during sexual activity was related to a higher frequency of crack use (*r* = 0.65, *p* < 0.001).

Among male MS, significant correlations were found between substance use and sexual functioning problems. No associations were identified between sexual function problems and the frequency of alcohol consumption or DAST. A decreased ability to experience sexual arousal was related to the frequency of cannabis consumption (*r* = −0.25, *p* = 0.03). Cocaine/crack use frequency correlated with masturbation frequency (*r* = 0.46, *p* = 0.05). LSD use frequency was found to be negatively correlated with the ability to achieve erection (*r* = −0.69, *p* = 0.01), while cannabis use frequency was found to be negatively correlated with the ability to maintain erection (*r* = −0.33, *p* = 0.01).

Regarding trauma-related factors, associations were identified between the severity of emotional neglect and decreased satisfaction with sexual life (*r* = −0.22, *p* < 0.001). The frequency of pain during sexual activity was associated with the frequency of currently occurring intrusions (*r* = 0.24, *p* = 0.01) and avoidance (*r* = 0.21, *p* = 0.02). The frequency of currently occurring hyperarousal and decreased satisfaction with sexual life (*r* = −0.25, *p* < 0.001) and ability to enjoy sexual activity (*r* = −0.25, *p* < 0.01).

A decreased ability to enjoy sexual activity was correlated with the severity of emotional abuse (*r* = −0.23, *p* = 0.03) and neglect (*r* = −0.26, *p* = 0.01). Additionally, the severity of emotional neglect was further associated with decreased satisfaction with sexual life (*r* = −0.28, *p* = 0.01), ability to maintain erections (*r* = −0.23, *p* = 0.03), and decreased satisfaction with orgasm intensity (*r* = −0.36, *p* < 0.001). Another correlation was identified between the severity of physical neglect and a decrease in satisfaction with orgasm intensity (*r* = −0.24, *p* = 0.02). A decreased satisfaction with sexual life was related to the frequency of occurring intrusions (*r* = −0.35, *p* = 0.02) and hyperarousal (*r* = −0.37, *p* = 0.01). Furthermore, a decreased ability to enjoy sexual activity was related to the frequency of occurring avoidance (*r* = −0.36, *p* = 0.02).

### 3.3 Prediction of compulsive sexual behavior

#### 3.3.1 Results of stepwise linear regression

The results of stepwise linear regression are presented in Table 6. Male sex, higher AUDIT scores, and frequency of currently occurring hyperarousal were significant predictors of higher CSBD scores. No significant moderation effects were found, indicating a potential interaction effect of the IES-R subscales on the relationship between AUDIT-/DAST- and CSBD scores.

**Table 6:** A final multivariate regression model with CSBD score as dependent variable (*N* = 157).

	B	SE	β	*T*	*p*	LLCI	ULCI	VIF
*Constant*	*9.068*	*2.140*		*4.237*	*<0.001*	*4.840*	*13.297*	
Hyperarousal (IES-R)	0.421	0.081	0.396	5.208	<0.001	0.261	0.580	1.235
AUDIT score	0.480	0.132	0.237	3.648	<0.001	0.220	0.740	1.041
Sex (male)	9.286	1.464	0.417	6.345	<0.001	6.394	12.177	1.063
Psychiatric diagnosis (yes)	1.735	1.534	0.079	1.131	0.260	−1.296	4.765	1.204

CSBD, Compulsive Sexual Behavior Disorder Scale; B, regression coefficient; SE, standard error; LLCI, lower level 95% confidence interval; ULCI, upper level 95% confidence interval; VIF, variance inflation factor. Residuals were not normally distributed (Shapiro–Wilk-Test *p* < 0.001). The White-test indicates homoscedasticity of residuals, χ^2^(63) = 123.98, *p* < 0.001. *F*(4, 152) = 23.57, *p* < 0.001. Adjusted *R*^2^ = 0.37.

#### 3.3.2 Results of path analysis

**Figure 4:**
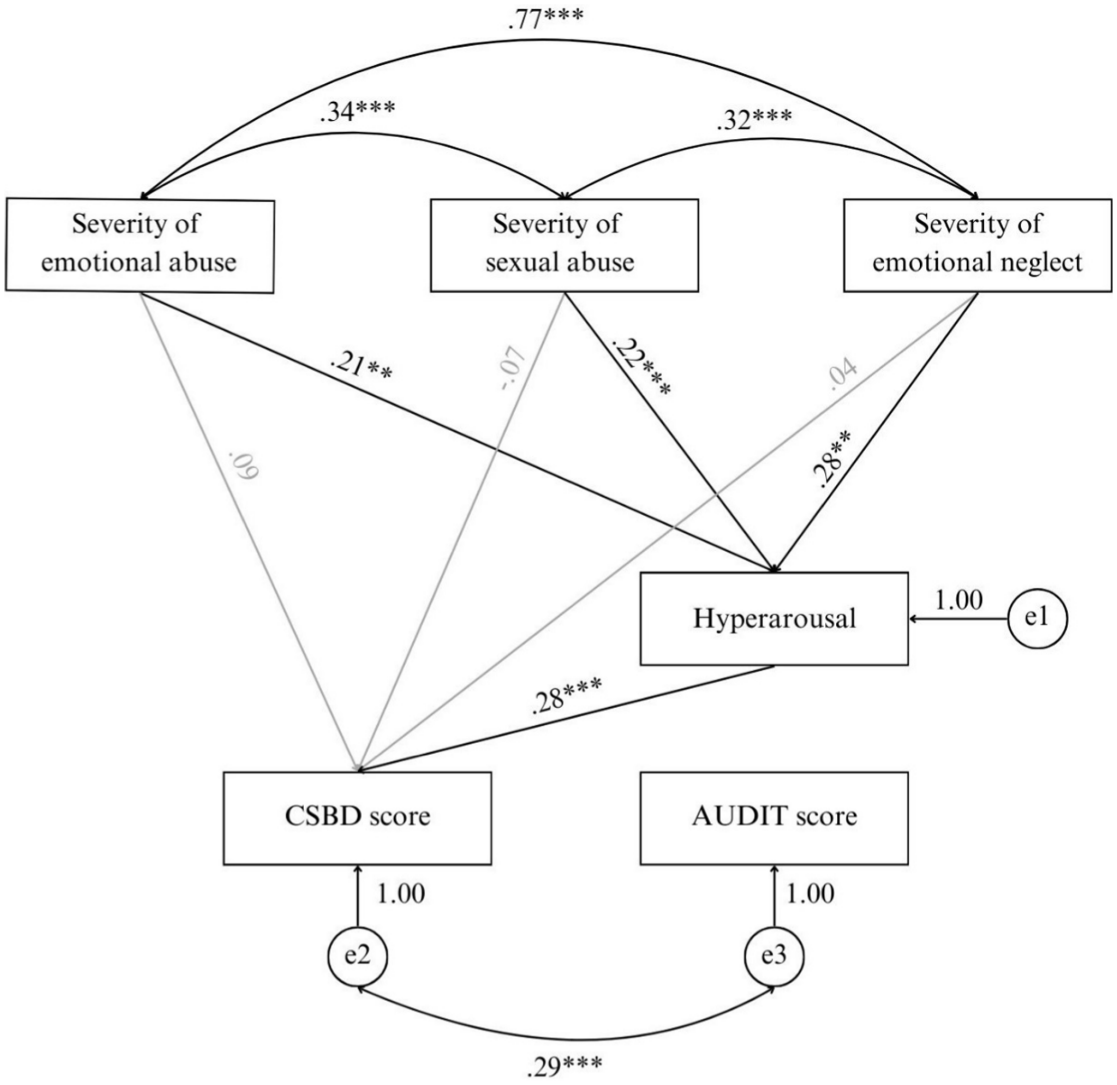
The final model of path analysis compulsive sexual behavior and alcohol use (*N* = 358). CSBD, Compulsive Sexual Behavior Scale; AUDIT, Alcohol Use Disorder Identification Test; *e*, residual. *** = *p* < 0.001. ** = *p* < 0.01. * = *p* < 0.05. Model fit: *χ^2^*(4) = 1.34, *p* = 0.86. RMSEA = 0.00, *90% CI* [0.00; 0.04]. CFI = 1.00.

The results of the confirmatory path analysis can be found in the Supplementary Table S2. Since DAST was not correlated or identified as a significant predictor of CSBD score, path analyses were only conducted, including the AUDIT score. In the first step of the model specification, the insignificant paths were excluded. The final path model with standardized path coefficients (?) is illustrated in Figure 4. Indices indicate a good model fit, *χ^2^*(4) = 1.34, *p* = 0.86. RMSEA = 0.00, 90% CI [0.00; 0.04]. CFI = 1.00.

### 3.4 Prediction of sexual functioning problems

The results of all ordinal regression models with types of sexual functioning problems as dependent variables can be found in the Supplementary material.

#### 3.4.1 Prediction of sexual functioning problems among female medical students

Among female MS, the following significant predictors of higher frequencies in SFP were identified via ordinal regression models: lower values in the severity of emotional abuse as a predictor of higher frequencies in problems with achieving orgasm (*B* = −0.21, *p* = 0.03, *R*^2^ = 0.23) and orgasm intensity (*B* = −0.36, *p* = 0.00, *R*^2^ = 0.29). Childhood physical abuse severity predicted a higher frequency of problems with orgasm intensity (*B* = 0.37, *p* = 0.04, *R*^2^ = 0.29). As the Nagelkerke’s *R*^2^ value was not within an acceptable range, the ordinal regression models of pain during sex (*R*^2^ = 0.19) among female MS could not be considered. See the results of all the calculated models with female sexual functioning problems as dependent variables in the Supplementary material. As AUDIT and DAST were not identified as significant predictors of SFP, no path analyses were calculated.

#### 3.4.2 Prediction of sexual functioning problems among male MS

None of the ordinal regression models examining the sexual functioning problems of male MS as a dependent variable were significant (see the results in the Supplementary material). Since no maximum likelihood estimation could be calculated for the models with the frequency of problems with getting and maintaining an erection as a dependent variable, the goodness-of-fit measure is not sufficiently reliable. Harmful alcohol/drug use was not found to be a predicting variable for any of the investigated SFPs. Thus, no path analyses were conducted to examine these relations.

## 4 Discussion

### 4.1 Summary of results

The objectives of this study were to (1) provide a first overview of the frequencies of CSB and SFP among MS, (2) identify predictors of CSB and SFP among MS related to substance use and trauma-related factors, and (3) investigate the interaction effects between CSB/SFP, substance use, and trauma-related factors.

One-third of female MS reported a decreased sexual desire and problems with achieving orgasm. A higher frequency of premature ejaculation was reported by around a fifth of male MS. Other sexual functioning problems, as well as CSB, were rarely reported in the sample. More intense CSB was predicted by the harmfulness of alcohol use, the frequency of post-traumatic hyperarousal, and the male sex. Path analyses revealed significant effects of emotional and sexual abuse as well as emotional neglect on the intensity of currently occurring post-traumatic hyperarousal, which in turn affected the intensity of CSB, which conclusively covariates with the harmfulness of alcohol use. The model should not be rejected (considering the χ^2^-test and 90% CI of RMSEA, which suggests a good model fit), but however, considering RMSEA and CFI, it seems that with this data basis, the model is not able to evaluate the effects demonstrably. Possible reasons for that are the small effect sizes and sample size.

Among female MS, sexual function was negatively related to the frequency of cannabis, cocaine/crack, and speed use (however, the use frequencies of these substances were reported to be low in general), but also to the severity of emotional neglect and the intensity of currently occurring post-traumatic stress symptoms. Whereas lower levels of emotional abuse severity and higher levels of physical abuse severity predicted problems related to orgasm experience among female MS, no predictors of sexual functioning problems were identified among male MS. Thus, regression and correlation analyses could not provide a basis for conducting path analyses investigating the relationships between SFP, substance use, and trauma-related factors.

### 4.2 Implications

The cut-off value for compulsive sexual behavior, which indicates a high risk of having CSBD, was exceeded by 3.1% of MS, which is slightly lower than the general CSBD prevalence estimates for Germany (Briken et al., 2022) and worldwide (Bőthe et al., 2023). More intense CSB is predicted by the male sex (which is consistent with most studies on CSB among varying samples (Grubbs et al., 2020), harmful alcohol use, and current persisting hyperarousal as a post-traumatic stress reaction). The results of the path analyses suggest that the relationship between the intensity of currently occurring post-traumatic hyperarousal (significantly associated with the severity of emotional and sexual abuse) and the intensity of CSB is mediated by the harmfulness of alcohol use. Therefore, in combination with the results of regression calculations, MS who experienced more severe emotional trauma are at a higher risk of developing CSB as well as more harmful alcohol consumption. More research is essential to explore the interrelation between post-traumatic stress symptoms—especially hyperarousal—and CSB, while previous research indicates that CSB could be a strategy to cope with the occurring post-traumatic symptoms. Even though harmful alcohol use was associated with CSB in this study (and also consistent with Ballester-Arnal et al., 2020; Jepsen et al., 2024), no relation was found between the harmfulness of alcohol use and trauma-related factors. Thus, on the basis of this data, CSB appears to be predicted by post-traumatic stress independently from the harmfulness of alcohol use among MS.

The identified frequencies of SFPs among the male MS in this sample are roughly consistent with the results of current prevalence measurements of the same age group (age 18–25) (Briken et al., 2020). However, female MS in this study showed significantly higher frequencies of sexual arousal problems (30.7% vs. 16.8% in the comparative study) and lower frequencies of pain during sexual activity (8.3% vs. 16.2% in the comparative study) (Briken et al., 2020). Moreover, the frequencies of SFPs and childhood trauma exposure among MS in this study were significantly lower than those reported by Ignatova et al. (2022) and Shindel et al. (2008). The frequency of sexual (functioning) problems correlated with the consumption frequency of benzodiazepines among female MS, with cannabis and MDMA/ecstasy among male MS, and with cocaine/crack, speed, and AUDIT among both sexes.

Among female MS, less severe exposure to emotional abuse during childhood predicted higher frequencies of problems with achieving orgasm and orgasm intensity. These findings may seem surprising at first glance, as they contradict those of previous studies (Lutfey et al., 2008; Weiss et al., 2023). However, it should be considered that potential protection and resilience factors, such as, e.g., family cohesion, social support, high self-esteem, and low rumination (Fritz et al., 2018), were not examined. Thus, it remains unclear whether these associations are mediated or moderated by these factors. Moreover, there are diverse potential reasons for the impairment of the orgasmic experience. Our results indicate a difference in the frequency of experienced orgasm between male and female MS, which corresponds to the *gender orgasm gap*. This phenomenon describes a gap in orgasm frequency and quality between men and women (Döring and Mohseni, 2022). From a psychosocial perspective, power relationships dominate heterosexual intercourse, with penetrative sex and coitus at the center of heterosexual activities (Döring and Mohseni, 2022; Wade, 2016; Frith, 2013). According to Wade et al. (2005), “It [the gender orgasm gap] is […] strongly impacted by social forces that privilege men’s pleasure over women’s, an ignorance about the clitoris, a prioritization of men’s pleasure, the gendered sexy/sexual binary, and a coital imperative.” This gap also extends to the operationalization of measuring female orgasms. When examining the items of the Sexual Behavior Questionnaire (SBQ), it is difficult to believe that it adequately represents the embodied experience of female orgasm (Frith, 2013; Jannini et al., 2012). There is a need to incorporate the results and further research of qualitative feminist scholars (Frith, 2013) into a methodological-mixed research design to develop an improved method of measurement. This also implies the need for female-led science in these topics to provide an appropriate representation.

The most frequently consumed substances among MS were alcohol, cannabis, and amphetamines (ecstasy/MDMA, speed). As in most cases, the consumption pattern of illegal drugs seems unremarkable from a therapeutic perspective. Approximately one-third of our sample showed indications of harmful alcohol use, which is consistent with the findings of most previous research (Hoff et al., 2023; Jackson et al., 2016; Gajda et al., 2021; Gignon et al., 2015). While interpreting the significant associations found between AUDIT and CSBD in this sample, it is important to consider that these relationships may be moderated or mediated by various mental health issues. Previous studies have shown that academic stress (Sheehama et al., 2022), exhaustion, and financial concerns (Jackson et al., 2016; Dahlin et al., 2011) are associated with higher AUDIT scores among MS. Respective effects may be expected concerning further individual factors such as age, gender, specialty, and career stage (Wilson et al., 2022). However, it is widely recognized that MS are at high risk of experiencing increased levels of stress, depression, and anxiety symptoms (Dyrbye et al., 2006). These symptoms are also commonly associated with CSB (Schultz et al., 2014; Ciocca et al., 2022; Walton et al., 2017; Reid et al., 2014) and SFP (Gonçalves et al., 2023; Hamilton and Julian, 2014; Velurajah et al., 2022). Previous research suggests that the presence of sexuality-related problems can be a risk factor for developing and maintaining harmful substance use, which is especially important to emphasize in this connection, as physicians are a vulnerable group for developing problematic substance use patterns and substance dependencies (Dyrbye et al., 2006). As consumption patterns manifest in substance use disorders over time, affected physicians report various barriers to seeking help for substance use disorders, such as fear of stigma, denial of the disease, psychiatric comorbidities, as well as expected negative familial, social, professional, and economic consequences (Vayr et al., 2019; Rogoža et al., 2021). Stigma towards mental health problems and their treatment remains a serious problem within the profession. Especially younger physicians report more barriers to seeking help for mental health problems than older physicians do, such as confidentiality or an anticipated negative impact on career progression (Wijeratne et al., 2021).

The most frequently reported forms of childhood trauma exposure were emotional neglect and abuse, while female MS showed significantly higher levels of emotional abuse severity compared to male MS. Additionally, a significantly higher prevalence of emotional abuse (27.6%) was identified in MS compared to the general German population (10.2 to 18.5%) (Glaesmer, 2016; Witt et al., 2017). However, the prevalence of all other investigated traumatic childhood experiences in this sample is highly similar compared to estimates in the general German population (Glaesmer, 2016; Witt et al., 2017).

This study suggests that substance use, childhood trauma exposure, and currently persistent post-traumatic symptoms may be associated with problematic sexual behaviors among MS to some extent. Notably, harmful alcohol use, emotional abuse during childhood, and, among female MS, sexual arousal problems and orgasmic dysfunction seem to occur frequently. Therefore, these factors should be considered in mental health support measures targeting medical students.

In this context, it is necessary to (1) identify the primary mental health issue that may trigger other mental health problems, (2) develop a tailored, individually adapted case plan for psychosocial care, and (3) connect and refer cases to relevant institutions for counseling and support concerning substance use, sexuality-related problems, and trauma.

Addressing mental health challenges among MS generally requires psychological support, as highlighted by various studies on this population (Gupta et al., 2022; Vayr et al., 2019). However, nearly half of MS experiencing mental health problems report unmet related needs for support (Gupta et al., 2022). Therefore, it is crucial to create accessible, low-threshold, and anonymous mental health support tailored to the needs of MS, as these are currently lacking in most universities and affiliated institutions.

The provision of psychosocial support measures is important, but it is not the only factor that needs to be implemented to improve the mental health of MS. Moreover, it is crucial to start programs that primarily focus on the reduction of career-related stigma and anxiety already during medical studies, as these factors are well-known to be important causes of mental health problems among MS and physicians (Vayr et al., 2019; Wijeratne et al., 2021). Furthermore, several studies have found that changes to the MS-adapted curriculum and workload are necessary to decrease mental stress among MS (Slavin et al., 2014; Jestin et al., 2023; Del Carmen et al., 2019), which would be beneficial in preventing the development of maladaptive coping strategies (such as substance abuse) and resulting mental health consequences (such as problematic sexual behaviors).

### 4.3 Limitations

When interpreting the results of this study, it is important to consider that the data were collected from a single institution using a cross-sectional design. Therefore, the main findings cannot be directly generalized to all MS. It is also worth noting that in Germany, cannabis was classified as an illegal drug only until April 2024.

All relevant constructs were measured using well-validated questionnaires (AUDIT, DAST, CSBD-19, CTQ, and IES-R). However, no validated questionnaire currently exists that measures clinically significant sexual functioning problems according to DSM or ICD diagnoses.

It is necessary to investigate additional factors to further explore the relationships between problematic sexual behaviors, substance use, and trauma exposure, as well as their respective developmental pathways. Further studies should consider factors such as workload, elements of the medical curriculum, additional sexuality-related aspects (e.g., sexual experiences with current sexual partner(s)), and burdens in the participants’ individual life situations. Despite these limitations, the results of this study provide an initial overview of problematic sexual behaviors among MS and their associations with substance use and trauma exposure.

## 5 Conclusion

CSB and most measured SFPs were relatively infrequent within this sample of MS. However, the study found that harmful alcohol use, emotional abuse during childhood, and, specifically, among female MS, sexual arousal problems, or orgasmic dysfunction were found quite frequently. Alcohol use and the intensity of current persistent post-traumatic hyperarousal appear to predict CSB among MS, while SFP appears mainly related to the use frequency of illicit drugs.

In particular, among women, dissatisfaction with orgasmic intensity was predicted by less severe emotional abuse but more severe physical abuse during childhood. However, further research is needed to explore in greater depth the pathways and interactions between problematic sexual behaviors, substance use, and trauma.

It is essential to incorporate factors such as sexuality, substance use, and trauma into mental health support measures while also exploring their relationships with workload, career-related stigma, anxieties, and other curriculum-related factors. MS with trauma exposure should be considered a vulnerable group at higher risk for problematic sexual behaviors and harmful alcohol consumption.

## Data Availability

The datasets presented in this article are not readily available because the participants were ensured that the collected information would be processed solely within the scope of the research project and not shared with third parties. Requests to access the datasets should be directed to dennis.jepsen@medizin.uni-halle.de.
