# Supplementary material for: Compulsive sexual behavior, sexual functioning problems, and their linkages to substance use among German medical students: exploring the role of sex and trauma exposure
Source: Front Psychol. 2024 Dec 4;15:1423690. doi: 10.3389/fpsyg.2024.1423690 (PMC11661911; doi:10.3389/fpsyg.2024.1423690)
Supplement: Supplementary file 1 [file Data_Sheet_1.PDF]

Supplementary Material

Compulsive sexual behavior, sexual functioning problems, and linkages to substance use among German medical students – Exploring the role of sex and trauma exposure

Dennis Jepsen[1*], Tobias Luck[2], Christian Heckel[1], Jana Niemann[1], Kristina Winter[1, 3], Stefan Watzke[4]

[1]Institute of Medical Sociology, Interdisciplinary Center of Health Sciences, Medical Faculty, Martin-Luther-University Halle-Wittenberg, Germany

[2]Faculty of Applied Social Sciences, University of Applied Sciences Erfurt, Germany

[3]Institute for Social Medicine, Rehabilitation Sciences and Health Services Research, Nordhausen University of Applied Science, Germany

[4]Department of Psychiatry, Psychotherapy, and Psychosomatics, University Hospital Halle, Germany

*** Correspondence:**
Dennis Jepsen
dennis.jepsen@medizin.uni-halle.de

Keywords: Adverse childhood experiences, Addiction, Childhood trauma, Hypersexual behavior, Hyposexual behavior, Sexual dysfunction, Posttraumatic stress

Table 1. Skewness and kurtosis of relevant metric-scaled variables

Variable	Skewness	SE of skewness	Kurtosis	SE of kurtosis	N
CSBD score	1.54	.13	3.11	.26	340
AUDIT score	.93	.13	.36	.26	346
DAST score	2.94	.17	9.34	.34	208
Severity of emotional abuse (CTQ)	1.74	.14	3.15	.27	318
Severity of physical abuse (CTQ)	4.39	.14	22.51	.27	317
Severity of sexual abuse (CTQ)	5.38	.14	35.86	.27	316
Severity of emotional neglect (CTQ)	1.38	.14	1.74	.27	318
Severity of physical neglect (CTQ)	2.26	.14	6.43	.27	318
IES-R score	.82	.19	-.26	.37	170
Frequency of intrusions (IES-R)	1.12	.19	.37	.38	166
Frequency of avoidance (IES-R)	.67	.19	-.67	.38	164
Frequency of hyperarousal (IES-R)	1.21	.19	.53	.37	168

Note: SE = Standard error. CSBD = Score of Compulsive Sexual Behavior Scale. AUDIT = Score of Alcohol Use Disorder Identification Test. DAST = Score of Drug Abuse Screening Test. CTQ = Childhood Trauma Questionnaire. IES-R = Impact of Event Scale (revised). Skewness of -/+2 and kurtosis of -/+7 indicate normal distribution.

Table 2. Results of the path analysis regarding compulsive sexual behavior and alcohol use (N = 358)

Confirmatory model				
**Path**	**β**	**SE**	**z**	**p**
Severity of emotional abuse → Severity of physical abuse[1]	.60	.36	9.11	<.001
Severity of emotional abuse → Severity of sexual abuse[1]	.34	.46	5.68	<.001
Severity of emotional abuse → Severity of emotional neglect[1]	.77	1.00	10.74	<.001
Severity of emotional abuse → Severity of physical neglect[1]	.42	.51	6.90	<.001
Severity of physical abuse → Severity of sexual abuse[1]	.30	.19	5.11	<.001
Severity of physical abuse → Severity of emotional neglect[1]	.55	.37	8.54	<.001
Severity of physical abuse → Severity of physical neglect[1]	.36	.21	6.04	<.001
Severity of sexual abuse → Severity of emotional neglect[1]	.32	.48	5.38	<.001
Severity of sexual abuse → Severity of physical neglect[1]	.23	.28	3.98	<.001
Severity of emotional neglect → Severity of physical neglect[1]	.56	.57	8.62	<.001
Severity of emotional abuse → Intrusions	.29	.22	3.05	.00
Severity of physical abuse → Intrusions	.06	.39	.86	.39
Severity of sexual abuse → Intrusions	.26	.22	4.82	<.001
Severity of emotional neglect → Intrusions	.05	.23	.45	.66
Severity of physical neglect → Intrusions	.00	.26	.05	.96
Severity of emotional abuse → Avoidance	.40	.27	4.33	<.001
Severity of physical abuse → Avoidance	-.15	.47	-2.34	.02
Severity of sexual abuse → Avoidance	.36	.27	7.00	<.001
Severity of emotional neglect → Avoidance	.01	.27	.10	.92
Severity of physical neglect → Avoidance	.03	.31	.46	.65
Severity of emotional abuse → Hyperarousal	.19	.20	2.13	.03
Severity of physical abuse → Hyperarousal	.06	.34	.96	.34
Severity of sexual abuse → Hyperarousal	.21	.20	4.15	<.001
Severity of emotional neglect → Hyperarousal	.24	.20	2.52	.01
Severity of physical neglect → Hyperarousal	.05	.23	.84	.40
Severity of emotional abuse → CSBD	.02	.23	.19	.85
Severity of physical abuse → CSBD	.16	.42	2.25	.03
Severity of sexual abuse → CSBD	-.11	.27	-1.74	.08
Severity of emotional neglect → CSBD	-.02	.22	-.24	.81
Severity of physical neglect → CSBD	.07	.25	1.13	.26
Intrusions → CSBD	-.14	.11	-1.34	.18
Avoidance → CSBD	.17	.08	1.81	.07
Hyperarousal → CSBD	.28	.11	2.76	.01
CSBD → AUDIT[1]	.28	2.26	4.69	<.001
**Re-specified model**				
**Path**	**Estimate**	**SE**	**z**	**p**
Severity of emotional abuse → Severity of sexual abuse[1]	.34	1.55	-5.23	<.001
Severity of emotional abuse → Severity of emotional neglect[1]	.77	1.00	10.74	<.001
Severity of sexual abuse → Severity of emotional neglect[1]	.32	.48	5.38	<.001
Severity of emotional abuse → Hyperarousal	.21	.19	2.54	.01
Severity of sexual abuse → Hyperarousal	.22	.20	4.31	<.001
Severity of emotional neglect → Hyperarousal	.28	.18	3.32	.00

Severity of emotional abuse → CSBD	.09	.21	1.09	.28
Severity of sexual abuse → CSBD	-.07	.26	-1.12	.26
Severity of emotional neglect → CSBD	.04	.21	.47	.64
Hyperarousal → CSBD	.28	.09	3.53	<.001
CSBD → AUDIT[1]	.29	2.30	4.97	<.001

Note: *β* = standardized regression weights. *SE* = Standard error. *z* = z-value/ critical ratio. CSBD = Score of Compulsive Sexual Behavior Scale. AUDIT = Score of Alcohol Use Disorder Identification Test. [1]Covariances. Model fit of confirmatory analysis: $\chi^2(11) = 219.20$, $p < .001$. RMSEA = .23, 90% CI [.20; .26]. CFI = .73. Model fit of re-specified model analysis: $\chi^2(4) = 1.34$, $p = .86$. RMSEA = .00, 90% CI [.00; .04]. CFI = 1.00.

Table 3. Ordinal regression with frequency of decreased sexual desire among female medical students as dependent variable

	B	SE	df	p	CI-95%
AUDIT	**-0.18**	**0.07**	**1**	**.02**	**-.32; -.04**
DAST	.09	.09	1	.31	-.09; .27
Frequency Intrusions	.03	.06	1	.59	-.08; .14
Frequency avoidance	-.04	.04	1	.29	-.12; .04
Frequency hyperarousal	-.01	.06	1	.82	-.13; .10
Severity emotional abuse	-.14	.10	1	.16	-.35; .06
Severity physical abuse	-.13	.18	1	.46	-.47; .21
Severity sexual abuse	.09	.12	1	.45	-.14; .31
Severity emotional neglect	.07	.10	1	.46	-.12; .27
Severity physical neglect	.06	.12	1	.62	-.18; .30
Present somatic disease (yes)	.19	.62	1	.75	-.1.02; 1.41
Present psychiatric disease (yes)	-.41	.69	1	.55	-1.76; .93

Note: AUDIT = Alcohol Use Disorder Identification Test score. DAST = Drug Abuse Screening Test score. *B* = Unstandardized regression coefficient. *SE* = Standard error. *N* = 74. Nagelkerkes R^2 = .24. Significant predictors are shown in bold type.

Table 4. Ordinal regression with frequency of decreased orgasm intensity among female medical students as dependent variable

	B	SE	df	p	CI-95%
AUDIT	-.02	.06	1	.81	-.14; .11
DAST	.04	.08	1	.66	-.13; .20
Frequency Intrusions	-.02	.05	1	.78	-.12; .09
Frequency avoidance	.04	.04	1	.28	-.03; .11
Frequency hyperarousal	.06	.06	1	.29	-.05; .17
Severity emotional abuse	**-.36**	**.11**	**1**	**.00**	**-.58; -.14**
Severity physical abuse	**.37**	**.18**	**1**	**.04**	**.02; .71**
Severity sexual abuse	-.20	.12	1	.10	-.43; .03
Severity emotional neglect	.04	.09	1	.71	-.15; .22
Severity physical neglect	.05	.11	1	.67	-.17; .27
Present somatic disease (yes)	.16	.59	1	.79	-1.00; 1.32
Present psychiatric disease (yes)	.93	.67	1	.17	-.38; 2.24

Note: AUDIT = Alcohol Use Disorder Identification Test score. DAST = Drug Abuse Screening Test score. *B* = Unstandardized regression coefficient. *SE* = Standard error. *N* = 73. Nagelkerkes R^2 = .29. Significant predictors are shown in bold type.

Table 5. Ordinal regression with frequency of pain during sexual activity among female medical students as dependent variable

	B	SE	df	p	CI-95%
AUDIT	.07	.07	1	.32	-.07; .20
DAST	.07	.09	1	.45	-.11; .24
Frequency Intrusions	.04	.05	1	.51	-.07; .14
Frequency avoidance	.05	.04	1	.23	-.03; .12
Frequency hyperarousal	.02	.06	1	.79	-.10; .13
Severity emotional abuse	-.07	.10	1	.46	-.26; .12
Severity physical abuse	.00	.18	1	.99	-.34; .35
Severity sexual abuse	-.06	.11	1	.59	-.28; .16
Severity emotional neglect	-.07	.09	1	.47	-.25; .12
Severity physical neglect	-.07	.12	1	.54	-.30; .16
Present somatic disease (yes)	-.32	.60	1	.60	-1.48; .85
Present psychiatric disease (yes)	.08	.66	1	.91	-1.23; 1.38

Note: AUDIT = Alcohol Use Disorder Identification Test score. DAST = Drug Abuse Screening Test score. *B* = Unstandardized regression coefficient. *SE* = Standard error. *N* = 73. Nagelkerkes R^2 = .19. No significant predictors were identified.

Table 6. Ordinal regression with frequency of decreased sexual desire among male medical students as dependent variable

	**B**	**SE**	**df**	**p**	**CI-95%**
AUDIT	-.14	.13	1	.31	-.40; .13
DAST	.26	.30	1	.38	-.32; .84
Frequency Intrusions	-.04	.24	1	.88	-.50; .43
Frequency avoidance	.10	.10	1	.35	-.11; .30
Frequency hyperarousal	-.23	.18	1	.20	-.59; .13
Severity emotional abuse	-.18	.29	1	.53	-.74; .38
Severity physical abuse	.32	.40	1	.44	-.48; 1.11
Severity sexual abuse	-.36	1.24	1	.77	-2.79; 2.07
Severity emotional neglect	.14	.26	1	.58	-.37; .66
Severity physical neglect	.39	.27	1	.15	-.14; .92
Present somatic disease (yes)	-1.13	1.55	1	.47	-4.16; 1.90
Present psychiatric disease (yes)	-.02	1.46	1	.99	-2.88; 2.83

Note: AUDIT = Alcohol Use Disorder Identification Test score. DAST = Drug Abuse Screening Test score. *B* = Unstandardized regression coefficient. *SE* = Standard error. *N* = 30. Nagelkerkes R^2 = .33. No significant predictors were identified.

Table 7. Ordinal regression with frequency of delayed ejaculation among male medical students as dependent variable

	B	SE	df	p	CI-95%
AUDIT	.30	.27	1	.27	-.23; .84
DAST	-1.35	.76	1	.07	-2.84; .13
Frequency Intrusions	-.20	.43	1	.64	-1.06; .65
Frequency avoidance	-.05	.18	1	.78	-.40; .31
Frequency hyperarousal	1.16	.75	1	.12	-.31; 2.62
Severity emotional abuse	.55	.49	1	.26	-.40; 1.51
Severity physical abuse	.15	.62	1	.81	-1.07; 1.37
Severity sexual abuse	-5.67	4.63	1	.22	-14.76; 3.41
Severity emotional neglect	-.16	.40	1	.68	-.95; .62
Severity physical neglect	-.28	.39	1	.47	-1.05; .48
Present somatic disease (yes)	4.40	3.00	1	.14	-1.47; 10.27
Present psychiatric disease (yes)	.86	2.20	1	.70	-3.46; 5.17

Note: AUDIT = Alcohol Use Disorder Identification Test score. DAST = Drug Abuse Screening Test score. *B* = Unstandardized regression coefficient. *SE* = Standard error. *N* = 30. Nagelkerkes R^2 = .75. No significant predictors were identified.

Table 8. Ordinal regression with frequency of premature ejaculation among male medical students as dependent variable

	B	SE	df	p	CI-95%
AUDIT	.62	.38	1	.11	-.13; 1.37
DAST	.51	.51	1	.32	-.49; 1.50
Frequency Intrusions	-.43	.45	1	.34	-1.32; .45
Frequency avoidance	.41	.27	1	.12	-.11; .93
Frequency hyperarousal	-.37	.31	1	.22	-1.00; .23
Severity emotional abuse	.39	.48	1	.42	-.55; 1.34
Severity physical abuse	-.05	.56	1	.93	-1.14; 1.04
Severity sexual abuse	-4.43	3.50	1	.21	-11.29; 2.44
Severity emotional neglect	-.76	.47	1	.11	-1.69; .17
Severity physical neglect	1.14	.60	1	.06	-.04; 2.32
Present somatic disease (yes)	8.84	7.61	1	.25	-6.07; 23.76
Present psychiatric disease (yes)	-1.76	2.33	1	.45	-6.32; 2.79

Note: AUDIT = Alcohol Use Disorder Identification Test score. DAST = Drug Abuse Screening Test score. *B* = Unstandardized regression coefficient. *SE* = Standard error. *N* = 30. Nagelkerkes R^2 = .80. No significant predictors were identified.

Table 9: Ordinal regression with frequency of decreased orgasm intensity among male medical students as dependent variable

	estimate	SE	df	p	CI-95%
AUDIT	.14	.14	1	.32	-.14; .42
DAST	-.10	.32	1	.75	-.72; .52
Frequency Intrusions	.33	.25	1	.19	-.16; .82
Frequency avoidance	-.13	.10	1	.22	-.33; .08
Frequency hyperarousal	.01	.16	1	.93	-.29; .32
Severity emotional abuse	-.21	.29	1	.46	-.78; .35
Severity physical abuse	-.11	.42	1	.80	-.93; .71
Severity sexual abuse	2.16	3.11	1	.49	-3.93; 8.25
Severity emotional neglect	.35	.28	1	.21	-.12; .89
Severity physical neglect	.12	.26	1	.66	-.40; .63
Present somatic disease (yes)	-2.42	1.86	1	.19	-6.06; 1.22
Present psychiatric disease (yes)	-1.49	1.67	1	.37	-4.76; 1.78

Note: AUDIT = Alcohol Use Disorder Identification Test score. DAST = Drug Abuse Screening Test score. *B* = Unstandardized regression coefficient. *SE* = Standard error. *N* = 30. Nagelkerkes R^2 = .75. No significant predictors were identified.
